# The Fgl2 interaction with Tyrobp promotes the proliferation of cutaneous squamous cell carcinoma by regulating ERK-dependent autophagy

**DOI:** 10.7150/ijms.66929

**Published:** 2022-01-01

**Authors:** Mei Zeng, Qingxiang Li, Junzhao Chen, Wenfu Huang, Jinhua Liu, Cuiyan Wang, Manni Huang, Hui Li, Shu Zhou, Miaoying Xie, Kang Zeng

**Affiliations:** 1 Department of Dermatology, Huizhou Municipal Central Hospital, Huizhou 516000, Guangdong, People's Republic of China.; 2 Department of Dermatology, Nanfang Hospital, Southern Medical University, Guangzhou 510515, Guangdong, People's Republic of China.

**Keywords:** Cutaneous squamous cell carcinoma, Fgl2, Tyrobp, proliferation, autophagy

## Abstract

Human fibroleukin 2 (Fgl2), a member of the fibrinogen superfamily, can cleave prothrombin to generate thrombin or is secreted in a soluble form as a new type of effector of Tregs with immunomodulatory functions. However, there is little research on the role of Fgl2 in cutaneous squamous cell carcinoma (CSCC) growth. We examined the expression of Fgl2 in samples from CSCC patients and CSCC cell lines. Then, the effect of Fgl2 on CSCC was evaluated* in vitro* and in animals. Regulation of autophagy by Fgl2 was explored in CSCC. Coimmunoprecipitation (Co-IP) and immunofluorescence colocalization experiments were conducted to identify the regulatory effect of Fgl2 on the downstream protein Tyrobp. Then, gain- or loss-of-function analyses and evaluation of Tyrobp expression were performed to validate its role in autophagy and proliferation promoted by Fgl2. Here, our study demonstrated that Fgl2 promoted the proliferation of CSCC cells *in vitro* and *in vivo*. Knocking down Fgl2 reduced CSCC cell proliferation and inhibited autophagy in CSCC. Mechanistically, Fgl2 interacted with Tyrobp and promoted ERK-dependent autophagy, resulting in the proliferation of CSCC cells. Our study suggested that Fgl2 could be a promising prognostic biomarker and useful therapeutic target for CSCC.

## Introduction

Cutaneous squamous cell carcinoma (CSCC) is one of the most common nonmelanoma skin cancers in humans, and its incidence is increasing year by year with the development of the ageing population 1, 2. CSCC must be detected early and treated in time; otherwise, the tumour is prone to metastasis, invasion and destruction of tissues, resulting in poor prognosis and even death 3. At present, the clinical treatment of CSCC is mainly based on early surgical treatment, but due to its insidiousness, patients often miss the best opportunity for surgical treatment, and patients with advanced CSCC have deep tumour infiltration, often accompanied by lymph node metastasis. Even with surgical treatment, the prognosis is still poor 4. The occurrence and development of CSCC is a multifactorial process involving gradual evolution. There are currently no effective standard targeted therapeutic drugs or exact targets for CSCC treatment. Therefore, studying the molecular mechanism of the occurrence and development of CSCC and finding new biomarkers or therapeutic targets for CSCC have become important issues that urgently need to be resolved in clinical and scientific research.

Human fibroleukin 2 (Fgl2) is a member of the fibrinogen superfamily and has two different structural forms 5. One is secreted-type Fgl2, which is the soluble form of Fgl2 and is mainly secreted by CD4 + CD25 + Foxp3 + regulatory T cells (Tregs) and tolerogenic CD8 + CD45Rclow Tregs 6, 7. Studies have shown that the secreted Fgl2 protein has an immunosuppressive function 8, 9. Fgl2 secreted by tumour cells can prevent the differentiation of special subgroups of CD103 dendritic cells and participate in the regulation of the immunosuppressive tumour microenvironment 10. The other form of the Fgl2 protein is membrane-type Fgl2, a type II transmembrane protein that integrates with phospholipids of the cellular membrane, which is expressed in endothelial cells, epithelial cells, macrophages and dendritic cells and exerts procoagulant activity 11. However, research on membrane-type Fgl2 protein is mainly focused on the corresponding biological behaviour of its ability to cleave prothrombin into thrombin, while little is known about its role in cancers 12. The expression of Fgl2 is significantly higher in many cancers, including colon cancer, oesophageal cancer, gastric cancer, breast cancer, lung cancer, and cervical cancer, but weakly expressed or not expressed in normal tissues adjacent to cancer 5. Recent studies have reported that Fgl2 is overexpressed in hepatocellular carcinoma and that knocking down Fgl2 leads to inhibition of proliferation, which suggests that Fgl2 expression on cancer cells might be involved in tumour growth or other malignant biological behaviours^13^. However, the functional roles of the Fgl2 protein in CSCC are still unclear.

In this study, we attempted to explore the potential involvement of Fgl2 in CSCC. We found that the expression of Fgl2 was significantly increased in CSCC cell lines compared with a normal skin cell line. Similarly, Fgl2 was upregulated in clinical CSCC tissues compared to normal tissues. Moreover, we found that Fgl2 promoted the proliferation of human CSCC cells by inducing autophagic activity. Fgl2-induced autophagy was mediated by the interaction of Fgl2 and Tyrobp, resulting in the activation of ERK. Taken together, these findings suggest that Fgl2 may be a novel potential molecular target for treating CSCC.

## Results

### Fgl2 expression is upregulated in CSCC tissues and CSCC cell lines

**Figure 1:**
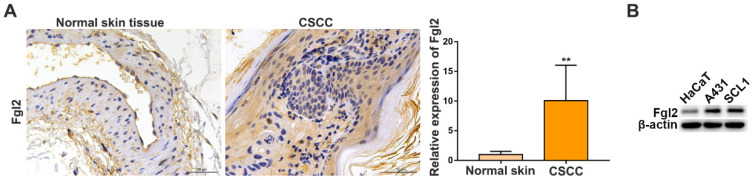
**Fgl2 is highly expressed in CSCC tissues and CSCC cell lines.** (A) Representative IHC staining of Fgl2 in samples from CSCC patients and normal skin controls (scale bar, 50 μm). (B) Western blot showing that Fgl2 was highly expressed in A431 and SCL1 cell lines compared with the human immortal keratinocyte line HaCaT.

To evaluate the clinical importance of Fgl2 in CSCC, we analysed Fgl2 expression by immunohistochemistry (IHC) in samples from CSCC patients and normal skin controls. The results showed that the expression of Fgl2 was significantly increased in the CSCC tissues compared with the normal skin tissues (Figure 1A). High levels of the Fgl2 protein were also observed in two CSCC cell lines, A431 and SCL1, compared with the human immortal keratinocyte line HaCaT (Figure 1B). Then, A431 and SCL1 cells were utilized as models to assess the function of Fgl2 in CSCC in subsequent experiments.

### Fgl2 promotes the proliferation of CSCC *in vitro*

**Figure 2:**
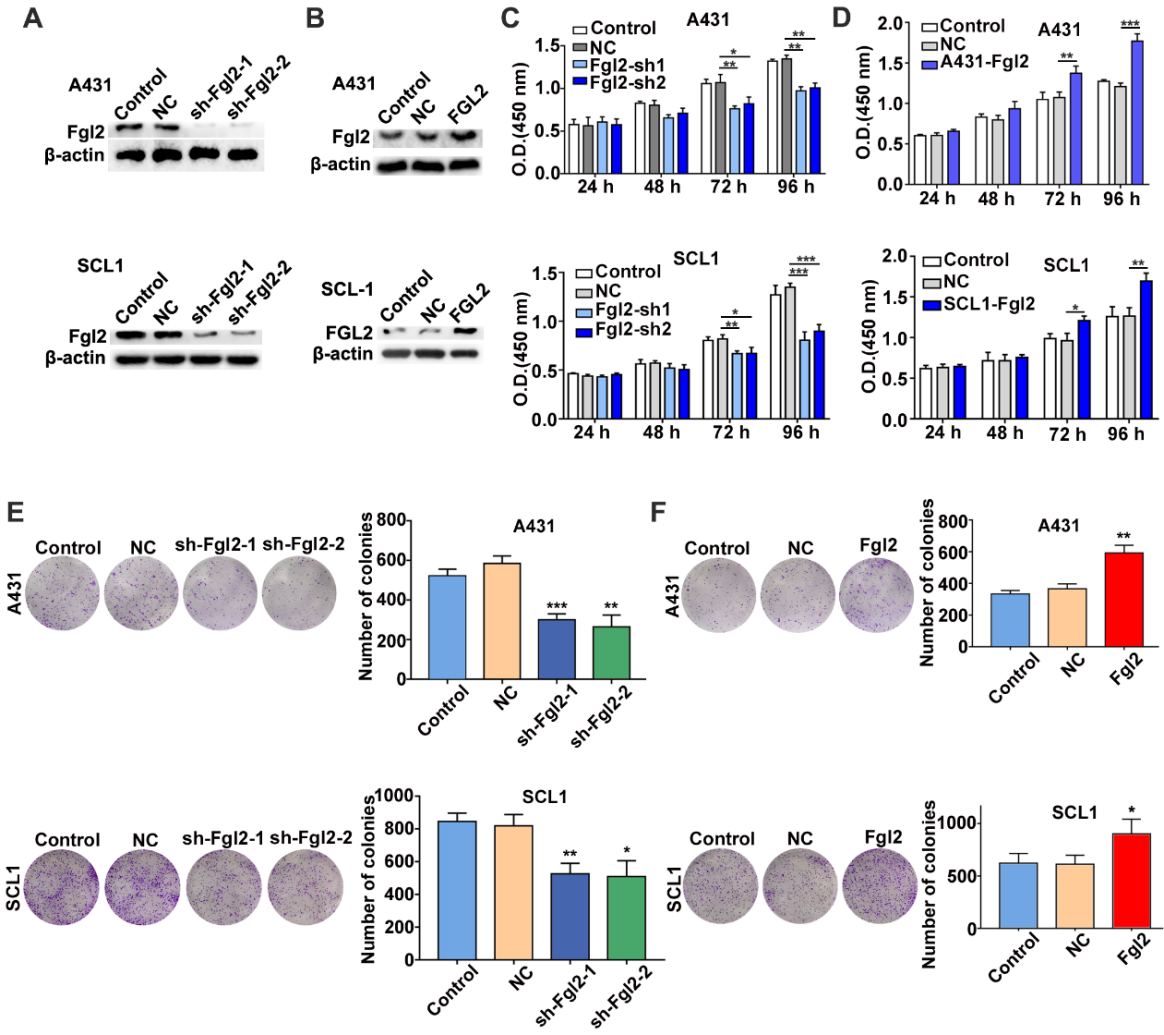
**Effects of Fgl2 on CSCC cell proliferation *in vitro*.** (A-B) Western blot showing Fgl2 levels in A431 and SCL1 cells with stably silenced Fgl2 expression (A) or overexpressing Fgl2 (B). (C) CCK-8 assays were used to determine the viability of CSCC cells with downregulated Fgl2 expression. (D) CCK-8 assays were used to determine the viability of CSCC cells with Fgl2 overexpression. (E) Colony formation assays were applied to determine the proliferation of CSCC cells with downregulated Fgl2 expression. (F) Colony formation assays were applied to determine the proliferation of CSCC cells overexpressing Fgl2. The data are presented as the mean ± SD of three independent experiments. *P < 0.05, **P < 0.01 and ***P < 0.001. sh-Fgl2-1: shRNA-Fgl2-1; sh-Fgl2-2: shRNA-Fgl2-2; NC: corresponding control vectors; Fgl2: cells with Fgl2-overexpressing.

To assess whether Fgl2 was functionally involved in the growth of CSCC, we developed A431 and SCL1 cells with stably silenced Fgl2 expression or overexpressed Fgl2 (Figure 2A and B). CCK-8 assays were performed to determine the effect of Fgl2 expression on CSCC cells. The results revealed that the proliferative capacity was decreased in the CSCC cells with downregulated Fgl2 expression, while proliferation was enhanced in the CSCC cells with Fgl2 overexpression (Figure 2C and D). Similarly, colony formation assays demonstrated that colony number was significantly reduced in the A431 and SCL1 cells with downregulated Fgl2 expression, while overexpressing Fgl2 increased the colony number of CSCC cells (Figure 2E and F). In summary, these results indicated that Fgl2 promoted proliferation in CSCC cells.

### Fgl2 promotes growth of CSCC *in vivo*

**Figure 3:**
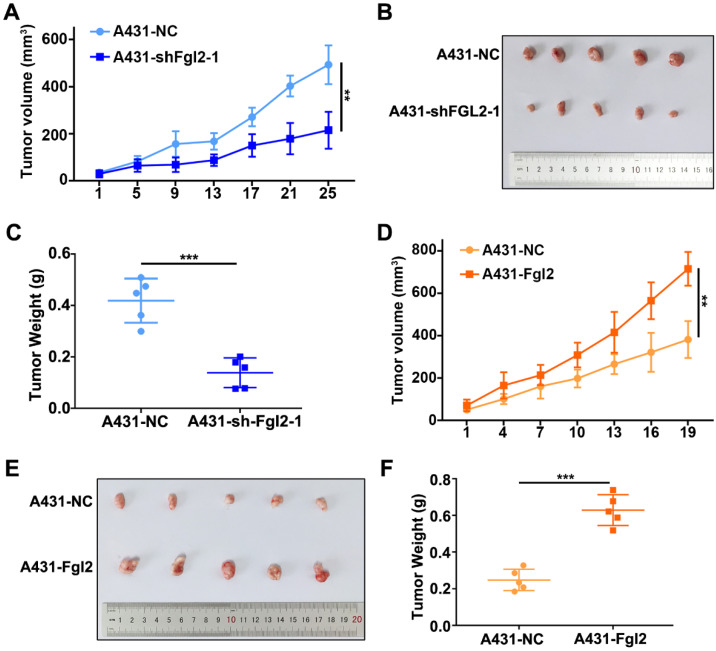
**Fgl2 strongly promoted CSCC growth* in vivo*.** (A and D) Growth curves of the Fgl2 knockdown groups or overexpression groups for tumour volumes were determined (**P<0.01). (B and E) Tumour formation of CSCC cells with downregulation of Fgl2 expression or overexpression. (C and F) Tumour weights were determined on the last day of animal experiments (***P<0.001).

To identify the effect of Fgl2 on tumour growth* in vivo*, we subcutaneously established mouse xenograft models using CSCC cells. The xenograft tumours derived from the A431 cells with stable silencing of Fgl2 expression had lower volumes and grew more slowly than the tumours derived from the control cells (Figure 3A-C). In contrast, Fgl2 overexpression significantly enhanced the tumour volumes and growth rates (Figure 3D-F). Fgl2 expression in subcutaneous xenograft tumours was verified by IHC (Figure S1A-B). Therefore, Fgl2 could enhance the growth of CSCC *in vivo*.

### Fgl2 is important for CSCC autophagy to promote proliferation

**Figure 4:**
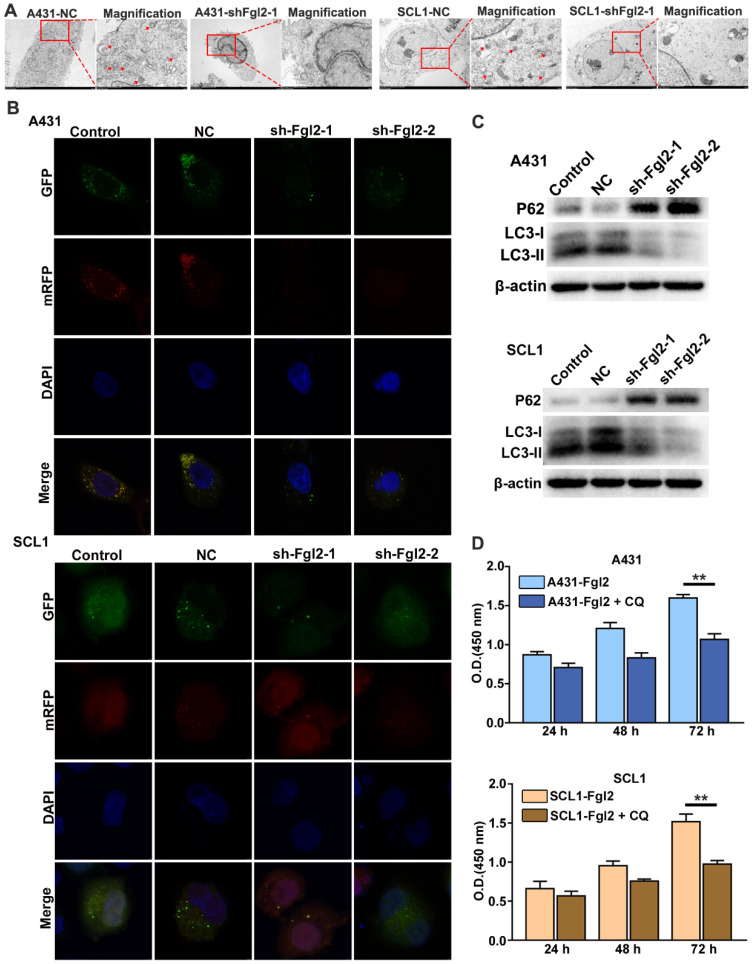
** Fgl2 is necessary for autophagy, contributing to CSCC cell proliferation.** (A) Autophagosomes were detected by transmission electron microscopy, and the magnified view shows autophagosomes indicated by red arrows. (B) Confocal laser scanning microscopy was used to examine the effect of downregulating Fgl2 expression on autophagy in CSCC cells. (C) Western blot analysis of the autophagy-associated proteins LC3-I/II and P62. (D) CCK-8 assays showed the attenuated proliferative effect of overexpressing Fgl2 in the presence of the autophagic inhibitor CQ (**P<0.01).

Autophagy is an adaptive process for uncontrolled and unlimited multiplication to maintain nutritional requirements by degrading damaged organelles and protein aggregates during uncontrolled and unlimited multiplication in cancer cells 14. It has been reported that cell autophagy plays an important role in the proliferation of CSCC cells 15. To investigate whether Fgl2 was involved in the autophagic process during CSCC cell proliferation, we examined the relationship between Fgl2 expression and autophagic activity in CSCC cells. The intracellular autophagosomes detected by transmission electron microscopy substantially decreased when Fgl2 expression was downregulated in CSCC cells (Figure 4A). Confocal laser scanning microscopy analysis showed a decreased mRFP-GFP-LC3 signal in the CSCC cells with silenced Fgl2 expression (Figure 4B). The expression of the autophagic markers LC3-II and p62 examined by Western blot assays also revealed autophagic activity along with the Fgl2 expression level in CSCC cells (Figure 4C). Furthermore, the proliferation-promoting effect of Fgl2 overexpression was significantly impaired when cells were treated with the autophagic inhibitor chloroquine (CQ) (Figure 4D). These data demonstrated that Fgl2 was necessary for autophagy in CSCC, contributing to cell proliferation.

### The interaction of Fgl2 and Tyrobp leads to CSCC cell proliferation

**Figure 5:**
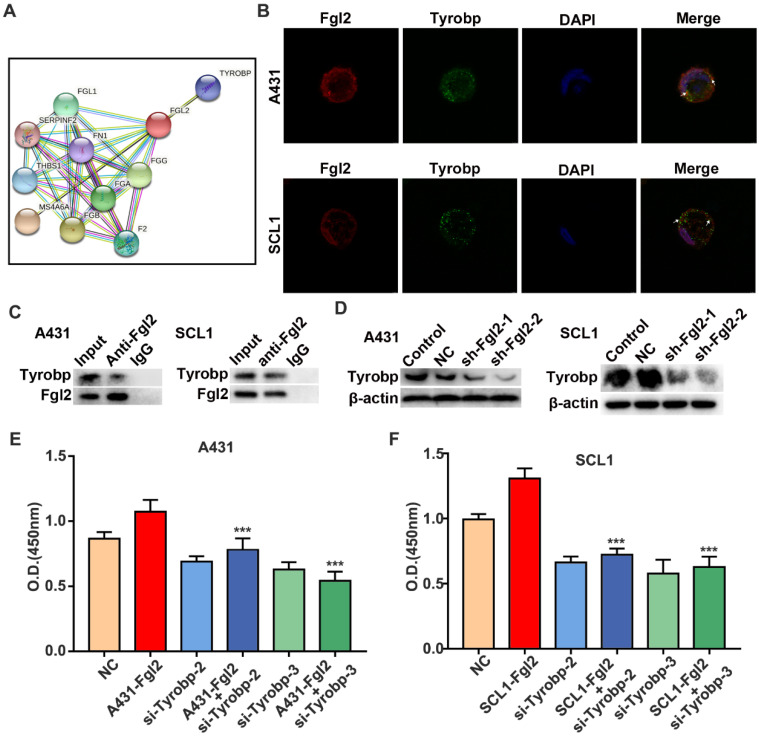
**Fgl2 interacted with Tyrobp, contributing to CSCC proliferation.** (A) The STRING database shows the protein-protein interaction (PPI) network of Fgl2. (B) Immunofluorescence for colocalization of Fgl2 and Tyrobp proteins in CSCC cells. (C) The interaction between Fgl2 and Tyrobp was analysed by Co-IP assays. Anti-Fgl2 antibody was used for IP, and the immunoprecipitates were examined by Western blots. (D) Tyrobp expression was analysed by Western blot analysis when Fgl2 was knocked down. (E and F) CCK-8 assays showed that downregulating Tyrobp expression attenuated the proliferation-promoting effect of overexpressing Fgl2 in A431 and SCL1 cells (***P<0.001).

To explore the mechanism of cell autophagy and proliferation regulated by Fgl2 in CSCC cells, we explored the molecular targets associated with Fgl2. The STRING database was used to build the protein-protein interaction (PPI) network. Two clusters were found (Figure 5A). One cluster was relevant to the coagulation function of Fgl2, which had been identified in previous studies 16, 17. Another cluster was Tyrobp, which is associated with multiple cell‐surface activating receptors. However, the role of Tyrobp in CSCC remains unclear. Subsequently, immunofluorescence colocalization analysis revealed colocalization of the Fgl2 and Tyrobp proteins in two CSCC cell lines (Figure 5B). To further identify whether the two proteins associate with each other, we performed a coimmunoprecipitation (Co-IP) assays of CSCC cells. The results showed that the Tyrobp protein level was associated with Fgl2 expression (Figure 5C). Notably, a reduction in Tyrobp expression was detected when Fgl2 expression was knocked down, which suggested the regulatory effect of Fgl2 on Tyrobp (Figure 5D). To determine whether the mechanism of Fgl2-promoted CSCC cell proliferation was associated with Tyrobp, we established CSCC cells transfected with siRNA targeting Tyrobp (Figure S2). CCK-8 assays revealed that downregulating Tyrobp expression significantly attenuated the proliferation-promoting effect of Fgl2 overexpression (Figure 5E and F). Thus, these results showed that Fgl2 promoted CSCC proliferation via the Tyrobp protein.

### Tyrobp induces ERK-dependent autophagy, leading to CSCC proliferation

**Figure 6:**
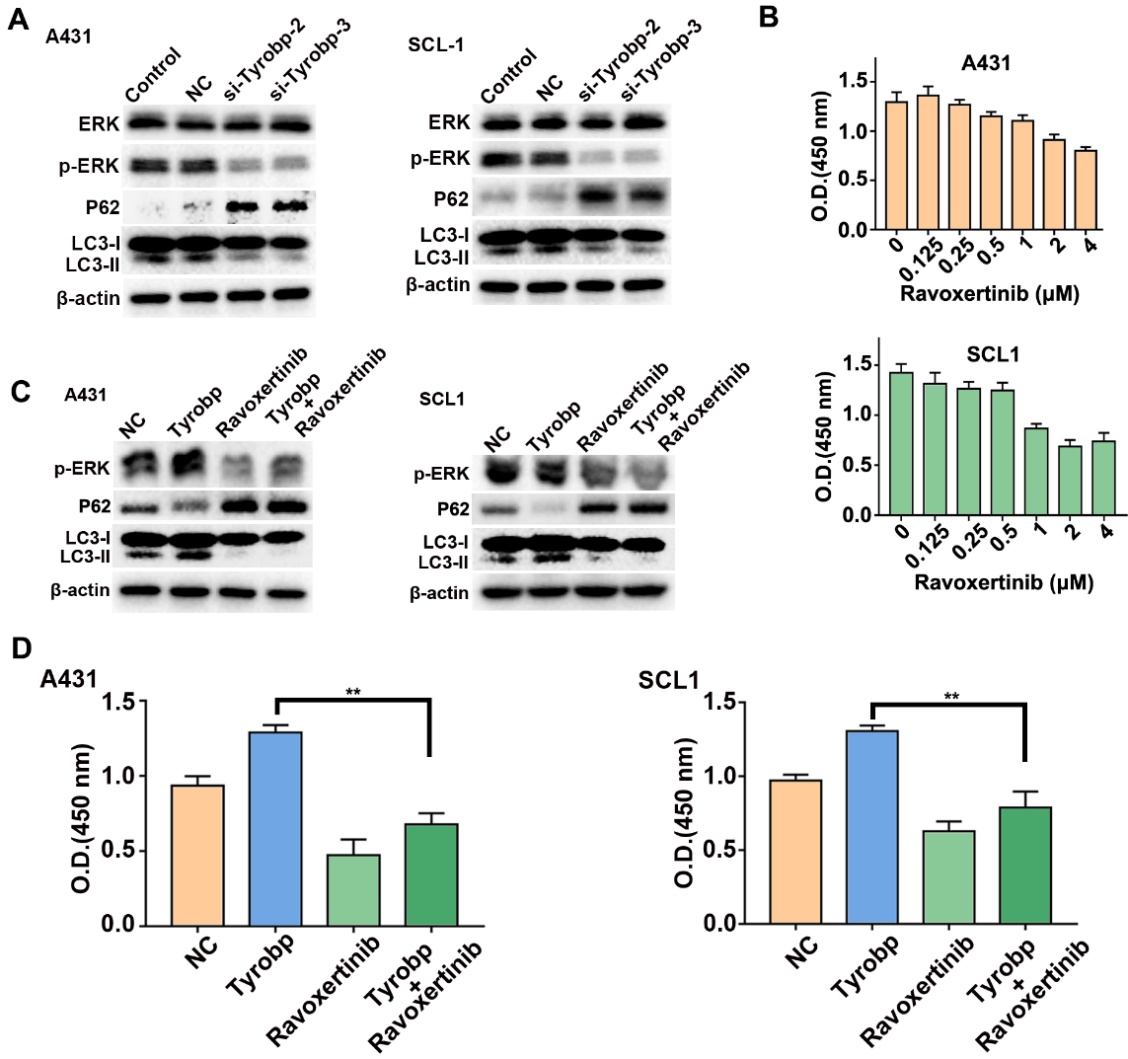
**Tyrobp induced ERK-dependent autophagy, resulting in CSCC cell proliferation.** (A) Western blot analysis of ERK activation and autophagic activity in CSCC cells with Tyrobp knockdown. (B) CSCC cells were incubated for 72 h in different concentrations of ravoxertinib and analysed by CCK-8 assays. (C) Western blot analysis of ERK activation and autophagic activity in CSCC cells overexpressing Tyrobp in the presence or absence of 2 μM ravoxertinib. (D) CCK-8 assays showed that the effect of proliferation induced by overexpressing Tyrobp was blocked in the presence of ravoxertinib.

Tyrobp is a signalling adaptor protein for cellular signal transduction and activates downstream signalling pathways, including the ERK pathway, which is involved in autophagic regulation 18, 19. To investigate whether Fgl2-induced autophagy was attributed to regulation of the ERK signalling pathway by Tyrobp, we detected ERK signalling and autophagic marker proteins in CSCC cells transfected with siRNA targeting Tyrobp. Knocking down Tyrobp significantly decreased the phosphorylation of ERK and autophagic activity in CSCC cells (Figure 6A). The ERK inhibitor ravoxertinib was applied to explore the effect of ERK on CSCC cell proliferation. As the concentration of ravoxertinib increased, inhibition of CSCC cell proliferation was observed (Figure 6B). Then, CSCC cells were transfected with plasmid to overexpress Tyrobp (Figure S3). The activation of ERK signalling and autophagy by overexpressing Tyrobp was significantly blocked by ravoxertinib (Figure 6C). Furthermore, proliferation promoted by overexpressing Tyrobp was significantly blocked when combined with ravoxertinib (Figure 6D). Therefore, these results revealed that Tyrobp targeted by Fgl2 promoted the proliferation of CSCC by regulating ERK-dependent autophagy.

## Discussion

**Figure 7:**
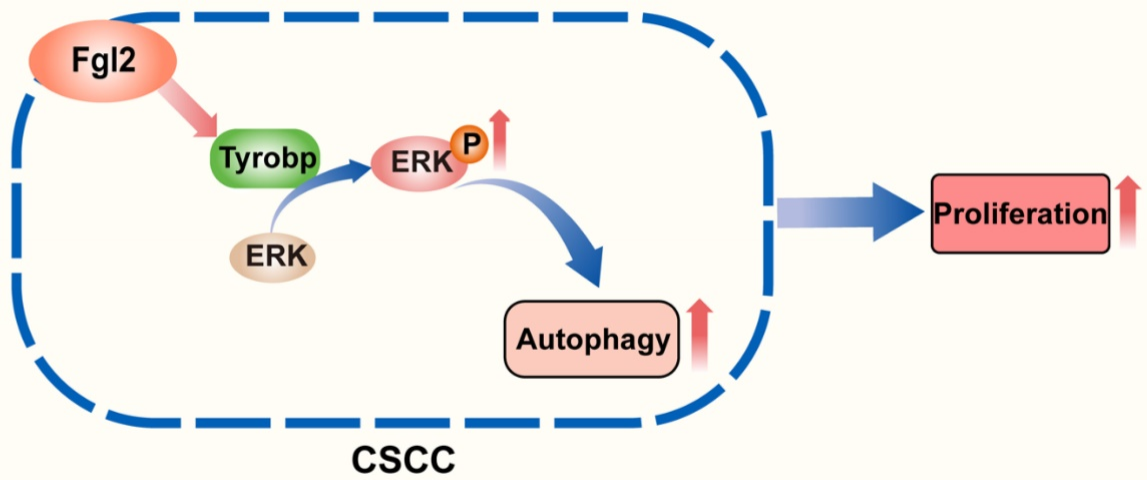
Schematic diagram showing that the interaction of Fgl2 with Tyrobp promoted the proliferation of cutaneous squamous cell carcinoma cells by regulating ERK-dependent autophagy.

The role of autophagy in pathogenic processes and antitumour therapy has been an issue of concern in recent years 20-22. The autophagic process of tumour cells was found to be an important survival- and growth-associated mechanism in many cancers in a previous study 23-25. However, the role and molecular mechanisms underlying autophagy in CSCC remain to be further clarified. In this study, we first revealed that Fgl2 interacting with Tyrobp promoted the proliferation of CSCC cells by regulating ERK-dependent autophagy (Figure 7).

To identify the function of Fgl2 in CSCC, we examined Fgl2 expression in samples from CSCC patients and two CSCC cell lines and found that CSCC tissues and the two CSCC cell lines highly expressed Fgl2 compared to normal skin tissues or cell lines. Then, we demonstrated that Fgl2 promoted CSCC proliferation and was necessary for autophagy in CSCC*.* To further elucidate the mechanism of Fgl2-related CSCC proliferation, we screened the potential molecular targets associated with Fgl2 in the STRING database, and Tyrobp was determined to be a novel downstream signal. Subsequent experiments showed that Fgl2 interacted with Tyrobp and that Tyrobp induced ERK-dependent autophagy, leading to CSCC cell proliferation.

Tyrobp, also named DNAX-activating protein of 12 kDa (DAP12), functions as an important adaptor protein mediating the intracellular signal transduction process of TREM2, SIRPβ1, CR3 and other receptors and participates in inflammation, phagocytosis, etc.^26^-^28^. In our study, a reduction in Tyrobp expression was detected when Fgl2 expression was knocked down. This finding might be because Fgl2 interacting with Tyrobp contributed to the stability of the Tyrobp protein and prevented it from being degraded by the proteasome. A previous study reported that Tyrobp acted as an immune-related signal transduction adaptin on the cellular membrane and played a significant role in the proliferation, survival, differentiation, and polarization of immune cells 29, 30. One important downstream target of Tyrobp is the ERK signalling pathway, which has been identified to regulate autophagic activity in many diseases 19, 31.

The ERK pathway is a classic antitumour therapeutic pathway, and many antitumour targeting drugs have been developed for this pathway 32, 33. Recently, with further research on the ERK pathway, the effect on regulating autophagy was widely verified. Autophagy is a response to external stressors and internal needs in tumour cells and is responsible for the degradation of bulk superfluous and nutrient recycling 34, 35. Although the effect of autophagy on tumour growth is two-sided, more recent studies have indicated that autophagy facilitates tumour growth and progression 36, 37. However, the mechanism of autophagy activated by ERK and its role in antitumour therapy remain to be further researched. Previous studies showed that after the ERK pathway was activated, phosphorylated ERK protein could upregulate the expression of autophagic proteins, such as LC3, resulting in cell autophagic induction^38^, ^39^. In contrast, it was reported that the ERK signalling pathway could decrease the expression of lysosomal-associated membrane protein 1 (LAMP1) and lysosomal-associated membrane protein 2 (LAMP2) and then block the binding of autophagosomes and lysosomes, leading to inhibition of autophagosome degradation 40. In our study, we found that the autophagy induced by Fgl2 occurred in an ERK signalling-dependent manner in CSCC. The specific molecular mechanism by which ERK signalling is activated by Fgl2-stimulated autophagy in CSCC still needs to be further explored in future research. The ERK inhibitor ravoxertinib was applied in our study to explore the effect of ERK on autophagy and CSCC cell proliferation. Notably, we found that ravoxertinib had antitumour effects on CSCC, indicating its valuable prospects in the clinical treatment of advanced and inoperable CSCC.

In summary, our study demonstrated that Fgl2 promoted the proliferation of CSCC cells *in vitro* and *in vivo*. Knocking down Fgl2 reduced CSCC cell proliferation, while autophagy in CSCC was inhibited. The mechanism was identified: Fgl2 interacted with Tyrobp and then positively regulated ERK-dependent autophagy, resulting in CSCC cell proliferation. In addition, our study implied that ravoxertinib might be a potential therapeutic drug for CSCC. This study suggested that Fgl2 could be a promising prognostic biomarker and useful therapeutic target for CSCC.

## Materials and methods

### Clinical samples

A total of 60 CSCC patient tissues were available from Huizhou municipal central Hospital (Huizhou, China) between January 2008 and January 2019 (Supplementary table 1). A total of 60 normal skin controls were collected from maxilloface, trunk or limbs. The agreement of every subject was obtained, and the experimental protocols complied with the principles of the Declaration of Helsinki. The experiments were approved by the Ethics Committee of Huizhou municipal central Hospital.

### Cell lines

The human CSCC cell line A431 was purchased from Procell Life Science & Technology (Wuhan, China). A431 cells were maintained in Dulbecco's modified Eagle's medium (DMEM) supplemented with 10% foetal bovine serum. Another human CSCC cell line, SCL1, was purchased from Ek Bioscience (Shanghai, China). SCL1 cells were maintained in RPMI-1640 supplemented with 10% foetal bovine serum. Approximately 1% penicillin and streptomycin were added to the medium. Cells were cultured in an incubator at 37 °C with 5% CO_2_.

### Reagents

The antibody to Fgl2 was obtained from Novus Biologicals. Antibodies against Tyrobp, β-actin, LC3B, phospho-p44/42 MAPK (Erk1/2) and p44/42 MAPK (Erk1/2) were obtained from Cell Signaling Technology (Danvers, MA, USA). The secondary antibodies were purchased from Abcam. Cell Counting Kit-8 (CCK-8) was purchased from Dojindo (Kumamoto, Japan). The autophagic inhibitor CQ was purchased from Sigma-Aldrich (St. Louis, MO, USA). Ravoxertinib was obtained from MedChemExpress (New Jersey, USA).

### Immunohistochemistry (IHC)

Tissue samples were fixed in 4% paraformaldehyde and embedded in paraffin blocks. Four-micron-thick sections were cut and analysed for Fgl2 protein. Slides were incubated with specific antibodies against Fgl2 overnight. After the samples were washed with PBS, they were treated with secondary antibodies and then stained with diaminobenzidine. The results were visualized by the EnVision peroxidase system (Dako). A total of 60 CSCC patient tissues and normal skin controls were analysed. The analysis was done by using the ImageJ program to measure the staining intensity of the dyed parts of diaminobenzidine.

### Cell transfection

The lentiviral particles of Fgl2 and shFgl2 were designed by and purchased from GeneCopoeia, Inc. (Rockville, MD, USA). CSCC cells were transfected with lentiviral particles using serum-containing medium supplemented with polybrene. The viral supernatants were removed after 48 h of transfection. The knockdown or overexpression efficiencies were determined by Western blot assays. CSCC cells were transiently transfected with validated siRNAs targeting Tyrobp and with the corresponding negative control siRNA (GenePharma Co., Ltd., Jiangsu Province, China). Cells were transfected with siRNAs using Lipofectamine 3000 (Invitrogen) for 2 days, and the transfection efficiencies were determined by Western blot assays.

### Cell Counting Kit-8 (CCK-8) assay

Cell proliferation was assayed by the Cell Counting Kit-8 (CCK8) assay. For the cell proliferation assay, CSCC cells were seeded at approximately 5000 cells per well in 96-well plates. According to the manufacturer's protocol, the proliferation of CSCC cells was evaluated every 24 h. After incubation with 10 μl of CCK-8 for 2 to 4 h, the absorbance of different groups was examined at 450 nm.

### Colony formation assay

CSCC cells with stably silenced Fgl2 expression or overexpressing Fgl2 were seeded in six-well plates. Then, the cells were incubated in an incubator with 5% CO_2_ at 37°C. After two weeks, the supernatant medium was removed, and the cells were washed three times with phosphate-buffered saline. Then, the cells were fixed with 4% paraformaldehyde and washed three times with phosphate-buffered saline again. Finally, colonies of cells were stained with 0.1% crystal violet.

### *In vivo* study

The BALB/c nude mice used were purchased from BesTest Bio-Tech (Zhuhai, China) and maintained under pathogen-free conditions. CSCC cells were harvested and suspended in phosphate-buffered saline, and 1 × 10^7^ cells were subcutaneously injected to establish a xenograft model. The volume of the tumour was calculated by an ellipsoid volume formula (length × width^2^/2). All animal experiments were performed with the approval of the Ethics Committee for Animal Experimentation of Southern Medical University.

### Western blot

Cells were collected and washed with PBS and then incubated at 0°C for 30 min in cell lysis buffer. Equal quantities of protein were electrophoresed by SDS-PAGE and transferred to PVDF membranes. The membranes were blocked with 5% bovine serum albumin. Then, the membranes were incubated with primary antibody overnight at 4°C and with a secondary horseradish peroxidase-labelled secondary antibody. Finally, immune complexes were detected with enhanced chemiluminescence reagents.

### mRFP-GFP-LC3 analysis

Adenoviral particles of mRFP-GFP-LC3 were purchased from Hanbio (Shanghai, China). Cells were seeded in a dish for an easy wall-adhering laser scanning confocal microscope. After cells with stably downregulated expression of Fgl2 and the corresponding negative control (NC) were infected with mRFP-GFP-LC3 adenovirus, 4% paraformaldehyde was used to fix the cells. Then, nuclei were stained with DAPI for 5 minutes. Samples were visualized by laser confocal microscopy.

### Transmission electron microscopy

CSCC cells with stably downregulated expression of Fgl2 and the corresponding negative control (NC) were fixed with 2.5% glutaraldehyde containing 0.1 mol/L sodium cacodylate, treated with 1% osmium tetroxide, embedded in araldite and cut into thin sections. The thin sections were stained with uranyl acetate and lead citrate. Samples were examined with an HT7800 transmission electron microscope (Hitachi, Inc., Tokyo, Japan) at 80 kV.

### Statistical analysis

The data are presented as the mean ± S.D. Independent samples t-test or one-way ANOVA was applied to analyse the differences between groups. Differences with *P* values < 0.05 were considered statistically significant. *P* values < 0.05, *P* values < 0.01 and *P* values < 0.001 are indicated with *, ** and ***, respectively.

## Supplementary Material

Supplementary figures and table.

## Abbreviations

Fgl2: Human fibroleukin 2
CSCC: cutaneous squamous cell carcinoma
Co-IP: Coimmunoprecipitation
IHC: immunohistochemistry
CQ: chloroquine
DMEM: Dulbecco's modified Eagle's medium
CCK8: Cell Counting Kit-8

## References

[1] Rogers HW, Weinstock MA, Feldman SR, Coldiron BM (2015). Incidence Estimate of Nonmelanoma Skin Cancer (Keratinocyte Carcinomas) in the U.S. Population, 2012. JAMA dermatology.

[2] Kallini JR, Hamed N, Khachemoune A (2015). Squamous cell carcinoma of the skin: epidemiology, classification, management, and novel trends. Int J Dermatol.

[3] Waldman A, Schmults C (2019). Cutaneous Squamous Cell Carcinoma. Hematology/oncology clinics of North America.

[4] Cañueto J, Martín-Vallejo J, Cardeñoso-Álvarez E, Fernández-López E, Pérez-Losada J, Román-Curto C (2018). Rapid growth rate is associated with poor prognosis in cutaneous squamous cell carcinoma. Clin Exp Dermatol.

[5] Yu J, Li J, Shen J, Du F, Wu X, Li M (2021). The role of Fibrinogen-like proteins in Cancer. International journal of biological sciences.

[6] Li XL, Ménoret S, Bezie S, Caron L, Chabannes D, Hill M (2010). Mechanism and localization of CD8 regulatory T cells in a heart transplant model of tolerance. J Immunol.

[7] Shalev I, Liu H, Koscik C, Bartczak A, Javadi M, Wong KM (2008). Targeted deletion of fgl2 leads to impaired regulatory T cell activity and development of autoimmune glomerulonephritis. J Immunol.

[8] Chan CW, Chan MW, Liu M, Fung L, Cole EH, Leibowitz JL (2002). Kinetic analysis of a unique direct prothrombinase, fgl2, and identification of a serine residue critical for the prothrombinase activity. J Immunol.

[9] Marazzi S, Blum S, Hartmann R, Gundersen D, Schreyer M, Argraves S (1998). Characterization of human fibroleukin, a fibrinogen-like protein secreted by T lymphocytes. J Immunol.

[10] Yan J, Zhao Q, Gabrusiewicz K, Kong LY, Xia X, Wang J (2019). FGL2 promotes tumor progression in the CNS by suppressing CD103(+) dendritic cell differentiation. Nature communications.

[11] Yuwaraj S, Ding J, Liu M, Marsden PA, Levy GA (2001). Genomic characterization, localization, and functional expression of FGL2, the human gene encoding fibroleukin: a novel human procoagulant. Genomics.

[12] Li WZ, Yang Y, Liu K, Long R, Jin N, Huang SY (2019). FGL2 prothrombinase contributes to the early stage of coronary microvascular obstruction through a fibrin-dependent pathway. International journal of cardiology.

[13] Liu Y, Xu L, Zeng Q, Wang J, Wang M, Xi D (2012). Downregulation of FGL2/prothrombinase delays HCCLM6 xenograft tumour growth and decreases tumour angiogenesis. Liver international: official journal of the International Association for the Study of the Liver.

[14] Jacob JA, Salmani JMM, Jiang Z, Feng L, Song J, Jia X (2017). Autophagy: An overview and its roles in cancer and obesity. Clinica chimica acta; international journal of clinical chemistry.

[15] Claerhout S, Verschooten L, Van Kelst S, De Vos R, Proby C, Agostinis P (2010). Concomitant inhibition of AKT and autophagy is required for efficient cisplatin-induced apoptosis of metastatic skin carcinoma. Int J Cancer.

[16] Wang J, Sanmamed MF, Datar I, Su TT, Ji L, Sun J (2019). Fibrinogen-like Protein 1 Is a Major Immune Inhibitory Ligand of LAG-3. Cell.

[17] Della-Morte D, Beecham A, Dong C, Wang L, McClendon MS, Gardener H (2012). Association between variations in coagulation system genes and carotid plaque. J Neurol Sci.

[18] Bosco MC, Pierobon D, Blengio F, Raggi F, Vanni C, Gattorno M (2011). Hypoxia modulates the gene expression profile of immunoregulatory receptors in human mature dendritic cells: identification of TREM-1 as a novel hypoxic marker *in vitro* and *in vivo*. Blood.

[19] Cagnol S, Chambard JC (2010). ERK and cell death: mechanisms of ERK-induced cell death-apoptosis, autophagy and senescence. FEBS J.

[20] Akkoc Y, Peker N, Akcay A, Gozuacik D (2021). Autophagy and Cancer Dormancy. Frontiers in oncology.

[21] Suares A, Medina MV, Coso O (2021). Autophagy in Viral Development and Progression of Cancer. Frontiers in oncology.

[22] Shen W, Zhang W, Ye W, Wang H, Zhang Q, Shen J (2020). SR9009 induces a REV-ERB dependent anti-small-cell lung cancer effect through inhibition of autophagy. Theranostics.

[23] Shen W, Zhang X, Fu X, Fan J, Luan J, Cao Z (2017). A novel and promising therapeutic approach for NSCLC: recombinant human arginase alone or combined with autophagy inhibitor. Cell death & disease.

[24] Chen L, He M, Zhang M, Sun Q, Zeng S, Zhao H The Role of non-coding RNAs in colorectal cancer, with a focus on its autophagy. Pharmacol Ther. 2021: 107868.

[25] Wang Z, Shi X, Li Y, Fan J, Zeng X, Xian Z (2014). Blocking autophagy enhanced cytotoxicity induced by recombinant human arginase in triple-negative breast cancer cells. Cell death & disease.

[26] Ma J, Jiang T, Tan L, Yu JT (2015). TYROBP in Alzheimer's disease. Mol Neurobiol.

[27] Konishi H, Kiyama H (2018). Microglial TREM2/DAP12 Signaling: A Double-Edged Sword in Neural Diseases. Frontiers in cellular neuroscience.

[28] Tessarz AS, Cerwenka A (2008). The TREM-1/DAP12 pathway. Immunol Lett.

[29] Lanier LL (2009). DAP10- and DAP12-associated receptors in innate immunity. Immunol Rev.

[30] Kobayashi M, Konishi H, Takai T, Kiyama H (2015). A DAP12-dependent signal promotes pro-inflammatory polarization in microglia following nerve injury and exacerbates degeneration of injured neurons. Glia.

[31] Snyder MR, Nakajima T, Leibson PJ, Weyand CM, Goronzy JJ (2004). Stimulatory killer Ig-like receptors modulate T cell activation through DAP12-dependent and DAP12-independent mechanisms. J Immunol.

[32] Samatar AA, Poulikakos PI (2014). Targeting RAS-ERK signalling in cancer: promises and challenges. Nat Rev Drug Discov.

[33] Fang JY, Richardson BC (2005). The MAPK signalling pathways and colorectal cancer. The Lancet Oncology.

[34] Glick D, Barth S, Macleod KF (2010). Autophagy: cellular and molecular mechanisms. J Pathol.

[35] Levine B, Kroemer G (2019). Biological Functions of Autophagy Genes: A Disease Perspective. Cell.

[36] Levy JMM, Towers CG, Thorburn A (2017). Targeting autophagy in cancer. Nat Rev Cancer.

[37] Kimmelman AC, White E (2017). Autophagy and Tumor Metabolism. Cell metabolism.

[38] Kim JH, Hong SK, Wu PK, Richards AL, Jackson WT, Park JI (2014). Raf/MEK/ERK can regulate cellular levels of LC3B and SQSTM1/p62 at expression levels. Exp Cell Res.

[39] Gu J, Hu W, Song ZP, Chen YG, Zhang DD, Wang CQ (2016). Rapamycin Inhibits Cardiac Hypertrophy by Promoting Autophagy via the MEK/ERK/Beclin-1 Pathway. Frontiers in physiology.

[40] Sivaprasad U, Basu A (2008). Inhibition of ERK attenuates autophagy and potentiates tumour necrosis factor-alpha-induced cell death in MCF-7 cells. J Cell Mol Med.

