# Supplementary material for: The Fgl2 interaction with Tyrobp promotes the proliferation of cutaneous squamous cell carcinoma by regulating ERK-dependent autophagy
Source: Int J Med Sci. 2022 Jan 1;19(1):195–204. doi: 10.7150/ijms.66929 (PMC8692121; doi:10.7150/ijms.66929)
Supplement: Supplementary file 1 — Supplementary figures and table. [file ijmsv19p0195s1.pdf]

Supplementary information

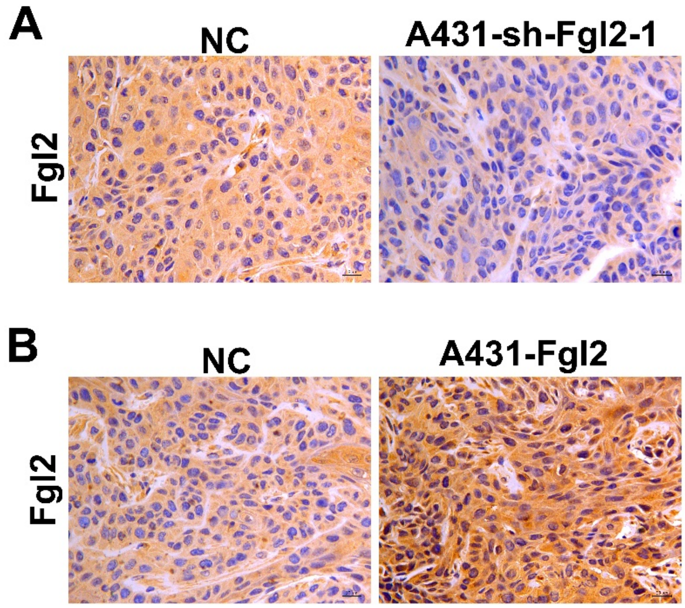

Figure S1. Fgl2 expression in subcutaneous xenograft tumours was verified by IHC. (A) Representative IHC staining of Fgl2 in subcutaneous xenografts of the Fgl2-silenced group or vehicle group. (B) Representative IHC staining of Fgl2 in subcutaneous xenografts of the Fgl2-overexpressing group or vehicle group.

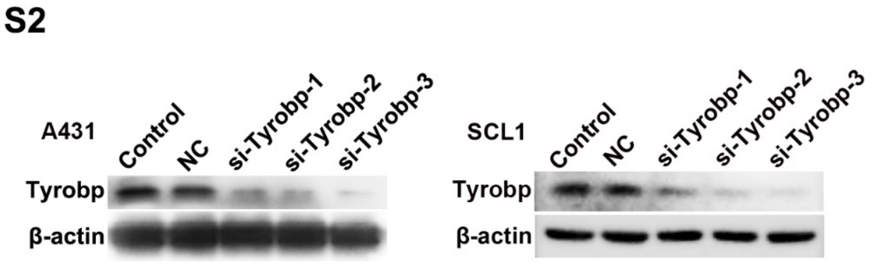

Figure S2. Western blot analysis determined Tyrobp levels in CSCC cells transfected with siRNA-Tyrobp.

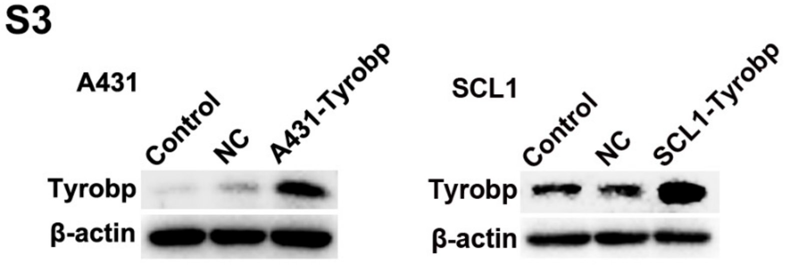

Figure S3. Western blot analysis determined Tyrobp levels in CSCC cells

overexpressing Tyrobp.

Supplementary table 1

Details of the clinical CSCC samples used in this study.

**Features**	**N**		
**Gender**	**Male**		**Female**
	**33**		**27**
**Differentiation status**	**Low**	**Moderately**	**High**
	**8**	**37**	**15**
**body location**	**Limbs**	**Trunk**	**Maxilloface**
	**28**	**5**	**27**
	**sun-exposed**	**sun-protected**	
	**55**	**5**	
